# Hypoglycemia Unawareness and Recurrent Severe Hypoglycemia in an Individual With Type 1 Diabetes Mellitus on Insulin

**DOI:** 10.1016/j.aace.2024.03.001

**Published:** 2024-03-06

**Authors:** Carlos Escudero, Alaa Husain, Amel Arnaout

**Affiliations:** 1 Faculty of Medicine, University of Ottawa, Ottawa, Ontario, Canada; 2 Division of Endocrinology and Metabolism, The Ottawa Hospital, Ottawa, Ontario, Canada

**Keywords:** type 1 diabetes mellitus, hypoglycemia, hypoglycemia unawareness, insulin, multidisciplinary care

## Abstract

**Background/Objective:**

Hypoglycemia unawareness is a complication of recurrent hypoglycemia that can complicate diabetes management and impact quality of life. We present the case of an individual with type 1 diabetes with hypoglycemia unawareness and recurrent severe hypoglycemia requiring emergency intervention.

**Case Report:**

A 55-year-old man with type 1 diabetes was referred for hypoglycemia unawareness and recurrent hypoglycemia with seizures. Over the prior 4 years he had >400 paramedic responses with 56 hospitalizations. Blood glucose levels ranged between 0.7 and 2.4 mmol/L during these episodes and presenting Hemoglobin A1c (HbA1c) was 4.6% (28 mmol/mol). He was taking insulin glargine daily and aspart with meals via insulin pens with no alternative etiology for his hypoglycemia was identified. The patient expressed difficulty with self-management, social instability, and limited appointment attendance. He was provided a continuous glucose monitor, educational support, and glycemic targets were broadened. After 6 months, HbA1c was 4.6% (28 mmol/mol) and he had 65 paramedic responses. A multidisciplinary team was organized for biweekly follow-up, community outreach, remote technological support, and psychological counseling. After 2 years, the patient had 2 emergency responses and HbA1c was 7.2% (55.2 mmol/mol).

**Discussion:**

Permissive hyperglycemia, educational interventions, and continuous glucose monitoring are validated strategies for prevention of hypoglycemia. Limiting hypoglycemia is crucial to restore hypoglycemia awareness, and in severe cases may require high intensity follow-up, community outreach, and psychosocial support.

**Conclusion:**

Hypoglycemia unawareness can complicate hypoglycemia prevention. Severe refractory cases are often multifaceted and may warrant a multidisciplinary approach to identify and target patient-specific needs.

**Highlights**

• Severe hypoglycemia unawareness can trigger recurrent hypoglycemic crises.
• Severe hypoglycemia unawareness may not respond to CGM and permissive hyperglycemia strategies.
• Multidisciplinary care may uncover patient-specific barriers to limit hypoglycemia.

**Clinical Relevance**

This is an extreme case of recurrent severe hypoglycemia in an individual with Type 1 diabetes mellitus (T1DM) and hypoglycemia unawareness with significant health care burden. Incorporating diabetes technologies, community outreach, frequent follow-up, and addressing psycho-social barriers may facilitate prevention of hypoglycemia in individuals with hypoglycemia unawareness.

## Introduction

Hypoglycemia, defined as a plasma glucose level below 4.0 mmol/L, is a common complication of insulin administration and is a limiting factor in optimizing glycemic management for individuals with diabetes. Hypoglycemia is characterized by autonomic and neuroglycopenic symptoms that may progress to syncope, convulsions, and coma.^1^ Recurrent hypoglycemia increases risk of hypoglycemia unawareness, cognitive impairment, and cardiovascular morbidity.^2^^,^^3^ Individuals with hypoglycemia unawareness develop increased risk of sudden onset severe hypoglycemia without preceding warning signs, which can restrict driving privileges, employment opportunities, impact interpersonal and social relationships, and even lead to death.^3^ Herein, we describe the case of an individual with type 1 diabetes mellitus (T1DM) experiencing hypoglycemia unawareness and recurrent severe hypoglycemic episodes necessitating almost daily Emergency Medical Services (EMS) interventions.

## Case Report

A 55-year-old man was referred to our clinic for management of recurrent severe hypoglycemia with frequent emergency intervention. His medical history included T1DM diagnosed at 24 years old and no other known conditions. He was taking insulin therapy and no other medications. In the first 5 years of disease, he experienced hypoglycemic episodes approximately 3 to 4 times per week that were preceded by autonomic warning signs such as confusion, diaphoresis, and tremors. Over subsequent years he developed hypoglycemia unawareness and increasingly frequent episodes of hypoglycemia with loss of consciousness and seizures for which he received emergency assistance. By the time he was assessed in our clinic, the patient had experienced 56 presentations to hospital and >400 EMS responses in the prior 4 years. The nadir of blood glucose (BG) levels ranged between 0.7 and 2.4 mmol/L during these episodes, and the presenting Hemoglobin A1c (HbA1c) was 4.6% (28 mmol/mol). His insulin regimen consisted of 30 units (U) of insulin glargine nightly, with insulin aspart 5U with breakfast, 3U with lunch, and 5U with supper. He self-monitored with glucometer 2 times per day, and no data on glycemic trends, diet or adherence to insulin regimen were available. Investigations conducted identified no contributory comorbidities, endogenous hyperinsulinism, endocrinopathies, organ dysfunction, or other drugs or toxins.

On initial assessment, the patient expressed difficulty regularly documenting blood sugars, counting carbohydrates and applying insulin correction scale, had occupational uncertainty with diet irregularity, limited understanding on treating low blood sugars, and family uncertainty around glucagon administration. We made a referral for government-funded continuous glucose monitor (CGM) coverage to facilitate self-monitoring, setting of hypoglycemia alerts, and to gather longitudinal continuous glycemic data. A diabetes nurse educator (DNE) and dietitian provided education on BG documentation and insulin self-administration, dietary advice including meal timing and carbohydrate ratios, and strategies for insulin self-administration and hypoglycemia treatment. We scheduled biweekly follow-up given the severity of the presentation.

**Fig. 1:**
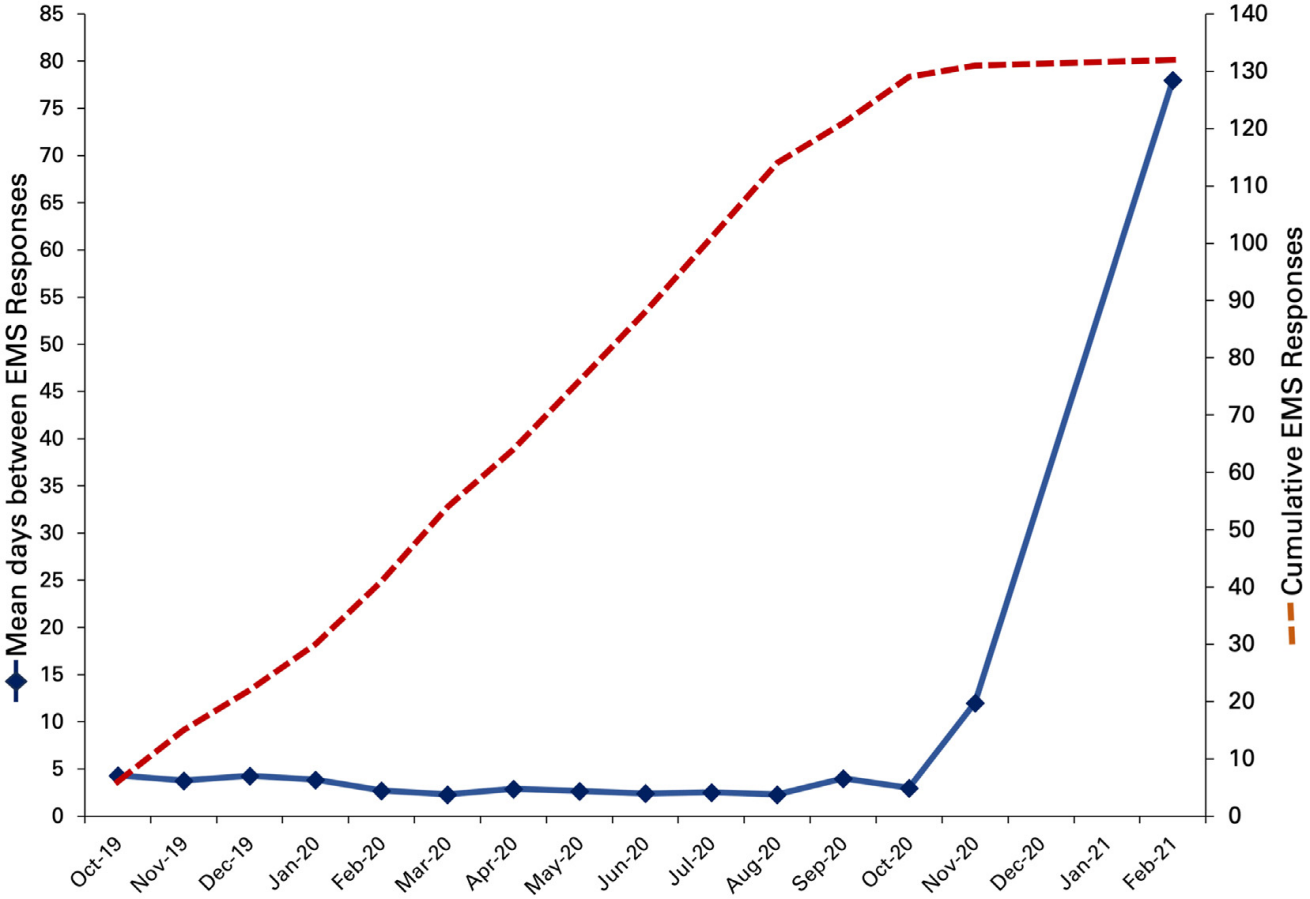
Episodes of hypoglycemia requiring emergency response. Trend of mean days between EMS responses (blue) and cumulative EMS responses (red) from initial presentation to our clinic until resolution of hypoglycemic crises with EMS response. *EMS* = Emergency Medical Services.

Over the subsequent 6 months the patient obtained a CGM, had monthly Endocrinologist appointments and weekly DNE follow-up. The insulin regimen was downtitrated to 28U glargine nightly, with 3U aspart with meals, targeting a fasting morning BG of 6 to 10 mmol/L and HbA1c of 7% to 8% (53-64 mmol/mol). The patient tracked hypoglycemic events and documented a food diary; however, did not obtain CGM sensors, share CGM data with the team, nor document insulin adherence, and did not attend multiple diabetes clinic appointments. Glycemic data available showed fasting morning BG was frequently in target range (6-10 mmol/L). Hypoglycemic events continued to occur daily in between meals, overnight, and sporadically throughout the day. After 6 months of follow-up, hypoglycemic events continued to occur daily and the patient had 65 EMS responses and 4 hospital presentations over 6 months (Fig. 1). A repeat HbA1c was 4.6% (28 mmol/mol).

Eleven months after initial consultation, a multidisciplinary care plan was created including our team, the DNE, family physician, the head of the local Emergency Department (ED), the municipal paramedic service, and a community paramedic program. We reviewed the patient’s medical history, hospitalizations, hypoglycemic events and management to date. Management changes made included downtitrating insulin to glargine 15U every morning and aspart 0 to 3U every meal with a sliding scale targeting BG 8 to 14 mmol/L 2 hours postprandially. We broadened CGM alert thresholds, organized CGM data sharing from the home, expedited applications for financial coverage for diabetes supplies, and increased follow-up frequency back to every 2 weeks. We enlisted the patient with a Community Paramedic Program to receive regular education and assistance in the community, including supplies to manage hypoglycemia at home, access to remote EMS support by phone, weekly community check-ins, and technological support for diabetes devices. He was seen by a psychologist for neuropsychiatric assessment, who detailed low health literacy, diabetes-related anxiety, occupational uncertainty, and burnout from his wife, the primary caregiver. In addition, the patient reported feeling shame and stigma around his condition during health care interactions.

Over the subsequent 7 months the patient shared CGM data more regularly with our team and had increased appointment attendance. He reported hypoglycemic events 1 to 2 times per week, had 10 EMS visits, and a 3-month period without hypoglycemic events requiring emergency response (Fig. 1). Follow-up frequency with our clinic was reduced to every 6 months. Two years after multi-disciplinary team (MDT) implementation, HbA1c was 7.2% (55.2 mmol/mol). The patient subjectively reported improved hypoglycemia awareness, had 2 hypoglycemic events with EMS visits and had zero presentations to hospital. There was ongoing follow-up with community supports.

## Discussion

Herein we describe the sustained resolution of hypoglycemic crises and recovery of hypoglycemia awareness in a patient with T1DM referred for recurrent hypoglycemia. Varied therapeutic interventions have been demonstrated to limit hypoglycemia in individuals with hypoglycemia unawareness, including personalized insulin regimens with permissive hyperglycemia, dietary and lifestyle changes, and CGM.^4^^,^^5^ CGM provides real-time BG readings, information on BG trends, and low glucose alarms to allow individuals to treat lows preventatively without relying on symptom onset. Uptake of CGM has been shown to improve patient autonomy and diabetes self-efficacy, reduce hypoglycemia frequency, and increase chances of restoring hypoglycemia awareness.^6^^,^^7^ National diabetes guidelines additionally recommend structured hypoglycemia education and prevention interventions such as the Blood Glucose Awareness Training Program.^8^

However, hypoglycemia unawareness can remain a therapeutic challenge because it is influenced by numerous factors that span physical, psychological, social and economic dimensions of well-being.^9^^,^^10^ We therefore conducted a comprehensive assessment through a MDT, which can aid in personalizing care for patients with complex care needs.^11^ We identified several interdependent barriers to standard hypoglycemia prevention. The delivery of structured diabetes and nutritional education had limited effectiveness due to low health literacy and dietary irregularity; the application of permissive hyperglycemia and CGM provided limited early benefit due to the patient’s social difficulty and low technological literacy; and maintaining hypoglycemia prevention practices was complicated by low appointment attendance in context of caregiver burnout, diabetes-related anxiety, and patient-reported stigmatization.

The patient’s low health literacy was addressed with personalized education on BG monitoring, insulin self-administration, and coordination with meals reinforced in the home setting.^12^ The patient’s dietary irregularity in the context of unstable occupational status was managed with the help of a nutritionist that provided personalized, financially accessible dietary options, and we addressed financial barriers through social assistance to obtain coverage for diabetes supplies including a CGM. Additionally, a psychologist helped to identify and manage life stressors, diabetes-related anxiety, and caregiver burnout through counseling and family support, which has been shown to independently limit hypoglycemia.^13^ We incorporated community supports to assist with technological difficulties such as data sharing, and to implement a structured plan to address hypoglycemia in the household with remote support, thus limiting need for EMS activation. Finally, we sought to limit stigmatization which has been shown, through improved rapport, to result in improved understanding of patient needs and improved outcomes.^14^

For severe cases of hypoglycemia unawareness requiring EMS support, multidisciplinary interventions as employed in this case may reduce health care costs by limiting EMS responses, ambulance transport, and hospital visits. In the first year post-MDT implementation, our patient required 96% less emergency responses and 92% less ED visits, saving an estimated $20 600 Canadian dollars in yearly health care expenditure.^15^^,^^16^ This case reflects literature that supports the use of community outreach programs to limit ED visits and reduce associated health care costs.^17^ However, this is an extreme example of hypoglycemia recurrence and emergency health care burden, and cost-benefit analyses are recommended on a case-by-case basis. Furthermore, our patient benefited from a resource-rich setting wherein social coverage for CGM, community outreach programs, and high intensity education are accessible. Alternative approaches in resource-limited settings may include high frequency provider follow-up, educational reinforcement in the community, and close BG monitoring with available technology. When available, CGM sensors may be selectively reserved for severe cases with greatest potential benefit.^18^

In this case, a multidisciplinary approach allowed us to identify and synchronously address multiple patient-specific barriers to care, resulting in improved appointment attendance, self-monitoring, data sharing, and reduced hypoglycemia frequency. In particular, use of CGM, high frequency community outreach, as well as reduced stigmatization and promotion of self-efficacy were central to this patient’s improvement. We propose that similar cases of recurrent hypoglycemia that are refractory to initial management may benefit from a multidisciplinary approach to identify and target patient-specific needs.

## Disclosure

The authors have no conflicts of interest to disclose.
